# The amount of ghrelin-immunoreactive cells in the abomasum and intestines of 13-14-week-old calves supplemented with Jerusalem artichoke flour alone or in combination with *Saccharomyces cerevisiae* yeast

**DOI:** 10.14202/vetworld.2022.1080-1086

**Published:** 2022-04-26

**Authors:** S. Jonova, A. Ilgaza, A. Ilgazs, M. Zolovs, L. Gatina

**Affiliations:** 1 Preclinical Institute, Faculty of Veterinary Medicine, Latvia University of Life Sciences and Technologies, Jelgava, Latvia; 2 Clinical Institute, Faculty of Veterinary Medicine, Latvia University of Life Sciences and Technologies, Jelgava, Latvia; 3 Department of Biosystematics, Institute of Life Sciences and Technology, Daugavpils University, Daugavpils, Latvia; 4 Statistics Unit, Riga Stradins University, Riga, Latvia

**Keywords:** calves, ghrelin, inulin, Jerusalem artichoke, *Saccharomyces cerevisiae*

## Abstract

**Background and Aim:**

The use of antibiotics in animals for disease prevention and productivity has been banned in the European Union since 2006. Possible alternatives can be used prebiotics, probiotics, and synbiotics. These compounds can improve feed digestion and absorption in the gastrointestinal tract with identical nutrient uptake, while imparting the feeling of satiety, which reduces the activity of ghrelin-immunoreactive (IR) cells. The number of studies performed on the activity of ghrelin-IR cells in ruminants is insufficient. In particular, there are few such studies in calves during the transition period from being a relatively monogastric animal to a ruminant. The present study aimed to evaluate the effect of Jerusalem artichoke flour (containing ~50% prebiotic inulin) and a new, commercially unavailable synbiotic (combination of Jerusalem artichoke flour and *Saccharomyces cerevisiae* strain 1026) on the amount of ghrelin-IR cells in the abomasum and intestines of 13-14-week-old calves.

**Materials and Methods:**

Fifteen crossbreed, Holstein Friesian and Red Holstein calves (*Bos taurus*) (32±4 days, 72.1±11.34 kg) were used. Calves were allocated into three groups: Control group (CoG, n=5) received the standard diet, prebiotic group (PreG, n=5) received 12 g of flour of Jerusalem artichoke (*Helianthus tuberosus*) per head containing 6 g of prebiotic inulin in addition to the standard diet, and synbiotic group (SynG, n=5) received a synbiotic in addition to the standard diet which consisted of two different products: 12 g of flour of Jerusalem artichoke per head containing 6 g of prebiotic inulin and probiotic 5 g of a yeast *S. cerevisiae* strain 1026. Feed additives were added to the concentrate once a day for 56 days. On days 1, 28, and 56, the live weight of the calves was determined. On day 56 of the experiment, three calves from each group were slaughtered. Histological samples were collected from the two parts of each calf abomasum: *Pars pylorica* and *pars fundalis* and the middle part of the duodenum and jejunum. Immunohistochemical tissue staining methods were used to detect ghrelin-IR cells.

**Results:**

The live weight of the slaughtered calves on day 56 was 115.3±21.73 kg in CoG, 130.0±17.32 kg in PreG, and 119.0±7.94 kg in SynG. Ghrelin-IR cells were more abundantly localized in the cytoplasm of the abomasum muscle gland cells in *pars fundalis* and *pars pylorica*, and to a lesser extent in the duodenum and jejunum. The number of ghrelin-IR cells in the abomasal fundic gland area was significantly higher in the CoG, than in the PreG and SynG (p=0.0001), while the difference between the PreG and SynG was not significant (p=0.700).

**Conclusion:**

The addition of Jerusalem artichoke flour and its combination with the yeast *S.cerevisiae* stain 1026 in calves resulted in a lower number of ghrelin-IR cells in the abomasum, duodenum, and jejunum and, although insignificantly, increased live weight (p=0.491), suggesting that calves in these groups with the same feed intake as the CoG had a better breakdown of nutrients, thus having a longer feeling of satiety.

## Introduction

The transition from milk feeding to roughage and concentrate feeding in calves’ lives is associated with various physiological and anatomical changes in the digestive tract, which is undergoing rapid microbial and structural modifications. This period causes additional stress in calves and may contribute to the development of various infectious diseases that may adversely affect their growth and development [1].

Today, with the rapid development of dairy farming, there is a growing need for healthier calves, which have better growth rates and are more productive. This is often achieved through the preventive use of antibiotics. However, the use of antibiotics in animals for disease prevention and productivity has been banned in the European Union since January 1, 2006, according to Regulation (EC) No. 1831/2003 of the European Parliament and of the Council [2].

Possible alternatives with similar properties are prebiotics, probiotics, and a combination of both synbiotics. The most significant effect of these additives is observed on the gastrointestinal microflora by promoting the growth of beneficial microorganisms. This, in turn, has a positive effect on the whole organism; animals show an increase in live weight, a lower number of pathogenic microorganisms in the digestive tract [3,4], and the structural and functional development of the rumen and intestine is promoted [5-9]. Prebiotics are non-digestible oligosaccharides (water-soluble carbohydrates) that advantageously influence the host by improving gut health and feed utilization and may reduce methane (CH_4_) production [4,10,11]. Inulin is one of the most well-known carbohydrates that are used as a prebiotic [12,13]. Probiotics, including *Saccharomyces cerevisiae* are microorganisms that have a broad range of beneficial effects such as regulation of intestinal microbial homeostasis, stabilization of gastrointestinal barrier function [14], and improvement of immunocompetence [15], resulting in enhanced body weight gain and feed efficiency [16]. Yeast also has the potential to change the fermentation process in the rumen by reducing the formation of CH_4_ gas [17]. Synbiotics are a combination of probiotics and prebiotics. These two products can have a synergistic impact that could be more significant than when used individually [18]. These feed additives can improve feed digestion and absorption in the gastrointestinal tract with identical nutrient uptake, while imparting the feeling of satiety, which reduces the activity of ghrelin-immunoreactive (IR) cells.

Ghrelin is a peptide of 28 amino acids in monogastric animals and 27 amino acids in ruminants [19], except in cattle, in which this peptide consists of 29 amino acids [20]. As in the stomach, the major molecular forms of ghrelin in the gut are n-octanoyl ghrelin and des-acyl ghrelin [21]. The main effect of ghrelin is to stimulate growth hormone secretion [19,21]. Ghrelin stimulates growth hormone release from pituitary cells by acting directly on the pituitary gland [20]. Growth hormone plays an important role in organ development [22]. Studies in lambs have shown that the release of increased levels of ghrelin from the abomasum contributed to its faster development. Therefore, ghrelin can be considered as being involved in the regulation of abomasal development [23]. Another important effect of ghrelin in the body is the regulation of appetite. Studies have shown that humoral factors that regulate appetite are found not only in the central nervous system but also in peripheral tissues [24,25]. Ghrelin is the only known peripherally produced and centrally active appetite-stimulating hormone that sends hunger signals from the stomach to the brain, controlling appetite and energy balance [19]. Ghrelin is produced in response to hunger and circulates in the blood, where it acts as a peripheral signal, indicating to the central nervous system that feed should be ingested [26,27].

In ruminants, ghrelin is mainly produced by the endocrine cells of the abomasal mucosa [28]. It has been reported that in adult ruminants and calves, the abomasum is the main site of ghrelin production. In the stomach, ghrelin-containing cells are found to a greater extent in the fundic gland area and a lesser extent in the pyloric gland area [21]. In both calves and cows, the number of ghrelin-producing cells in the gut gradually decreases from the duodenum to colon [21]. Minor amounts of ghrelin-producing cells are found in the pituitary, kidneys, lungs, placenta, testes, leukocytes, hypothalamus, and to a lesser extent in the adrenal glands, adipocytes, gallbladder, skeletal muscle, myocardium, skin, spleen, liver, ovaries, and prostate [29].

Many studies have been conducted on the activity of ghrelin-IR cells in monogastric animals [28,30], but the number of such studies in ruminants, whose stomach is never completely empty and whose digestive processes are continuous, is insufficient. In particular, there is a study on calves during the transition period from being a relatively monogastric animal to a ruminant [31].

Therefore, the present study was conducted to evaluate the effect of Jerusalem artichoke flour (containing ~50% prebiotic inulin) and a new, commercially unavailable synbiotic (combination of Jerusalem artichoke flour and *S. cerevisiae* strain 1026) on the amount of ghrelin-IR cells in the abomasum and intestines of 13-14-week-old Holstein Friesian and Red Holstein crossbred calves. At the same time, an effort was made to examine how these feed additives affect the live weight of calves and to indicate a possible correlation between live weight and the number of ghrelin-IR cells in the digestive tract of calves.

## Materials and Methods

### Ethical approval

All procedures performed in the present study were in accordance with ethical standards. The Council for Ethical Treatment of Animals, Latvia University of Life Sciences and Technologies approved this study (Nr. DzAĒP/2017/2). Three calves from each group were slaughtered at the end of the experiment to obtain samples of the gastrointestinal tract for histological examination. Following the ethical requirements to minimize the number of animals used in experiments, we chose to organize as small groups as possible (five animals per group).

### Study period and location

The study was conducted on a dairy cow farm from December 2017 to February 2018. The farm is located in Saldus district, Jaunlutriņi parish (latitude: N 56.8287° and longitude: A 22.4065°).

### Animals

Fifteen clinically healthy randomly selected Holstein Friesian and Red Holstein (*Bos taurus*) crossbreed calves with a mean age of 32±4 days and initial body weight of 72.1±11.34 kg were used in the present study. Throughout the study, the health status of the calves was evaluated by both the persons involved in the study and the farm veterinarian. No prolonged diarrhea, respiratory diseases, or other conditions were observed in the calves that could affect the study and the results obtained. The examination of the calves and the collection of all samples were conducted under the supervision of the farm veterinarian. All calves were housed in groups in a partly closed pen on the farm. After birth, all calves received colostrum, and for the next 5 days, calves received whole milk (3.5 L twice a day) and later the milk replacer in a dosage appropriate to their age and weight. Within the age of 32-64 days, calves received 8 L of milk replacer per day and a pre-starter diet without restriction (around 0.5 kg per calf/day). Within the age of 64-88 days, calves received approximately 1.5 kg of barley flour and 6 L of milk replacer per day. During the experiment, the calves had free access to hay and water. The chemical composition of the nutrients used in the study is presented in Table-1.

**Table 1:** Chemical composition of nutrients fed to calves during the study.

Composition (dry matter)	Pre-starter*	Milk replacer**	Hay	Barley flour
Crude protein, %	18.0	23.5	14.2	11.8
Crude fat, %	5.2	12.5	2.4	2.7
Crude fiber, %	7.0	3.5	32.1	3.3
Ash, %	6.5	9.0	5.6	4.4
Calcium, g/kg	14.0	9.0	3.3	4.4
Phosphorus, g/kg	7.0	7.0	3.0	4.0
Sodium, g/kg	6.0	4.0	0.2	16.2

* Supplements per 1 kg: Vitamin A, 35,000 IU; Vitamin D3, 3000 IU; Vitamin E, 250 mg; Vitamin B1, 13 mg; Vitamin B2, 6 mg; Vitamin B6, 5 mg; Vitamin B12, 40 µg, niacin, 36 mg; Ca-D-pantothenate, 20 mg; folic acid, 2 mg; Vitamin K3, 2 mg; choline chloride, 500 mg; biotin, 1000 µg; iron, 180 mg; copper, 25 mg; selenium, 0.4 mg; zinc, 120 mg; manganese, 120 mg, iodine, 3 mg; cobalt, 1 mg.

** Supplements per 1 kg: Vitamin A, 48,000 IU; Vitamin D3, 4000 IU; Vitamin E, 100 mg; Vitamin B1, 16 mg; Vitamin B2, 8 mg; Vitamin B6, 6 mg; Vitamin B12, 50 µg; niacin, 50 mg; Vitamin C, 250 mg; Ca-D-pantothenate, 25 mg; folic acid, 1 mg; Vitamin K3, 2 mg; choline chloride, 300 mg; biotin 200 µg; iron, 100 mg; zinc, 36 mg; copper, 4 mg; selenium, 0.4 mg; manganese, 48 mg; iodine, 1 mg

### Experimental design

Calves were allocated into three groups: Control group (CoG, n=5) received a standard diet, prebiotic group (PreG, n=5) received 12 g of flour of Jerusalem artichoke (*Helianthus tuberosus*) per head containing 6 g of prebiotic inulin (produced in Latvia, at the University of Latvia, Institute of Microbiology and Biotechnology) in addition to the standard diet, and synbiotic group (SynG, n=5) received a synbiotic in addition to the standard diet which consisted of two different products: 12 g of flour of Jerusalem artichoke per head containing 6 g of prebiotic inulin and probiotic 5 g of a yeast culture based on the *S. cerevisiae* strain 1026 (Yea-Sacc^®^, Alltech Inc., France).The yeast feed additive used in the study is registered for use in the countries of the European Union (EC Regulation No. 1109/2014). However, the synbiotic used in the study is not commercially available and was manufactured for this study. The flour of Jerusalem artichoke and synbiotic were added to the concentrate (pre-starter and later barley flour) once a day in the morning.

The duration of the experiment was 8 weeks (56 days). On days 1, 28, and 56 of the study, the live weight of the calves was determined by a recognized indirect live weight determining method using a special measuring tape Animeter (Albert Kerbl GmbH, Germany). Live weight can be determined by measuring the circumference of the chest just behind the elbows and reading the result on a tape measure. This method, which is based on the standard growth curves of cattle, is widely used to determine the live weight on farms [32].

At the end of the experiment, three calves from each group were slaughtered in a certified slaughterhouse following all humane slaughter guidelines. Histological samples were collected from the two parts of each calf abomasum: *Pars pylorica* and *pars fundalis*, as well as the middle part of the duodenum and the middle part of the jejunum. The obtained samples were rinsed with 0.9% sodium chloride solution and placed in bottles filled with 10% formalin (tissue-to-formalin ratio 1:10). The tissue samples were fixed in 10% formalin solution for at least 48 h. Immunohistochemical tissue staining methods were used to detect ghrelin-IR cells. The staining of IR cells was performed using the streptavidin-biotin complex (Dako REAL™ EnVision™ Detection System, Peroxidase/DAB+, Rabbit/Mouse, Agilent Technologies, USA). The examined tissue samples were applied to silane-coated slides (HistoBond®+, Paul Marienfeld GmbH and Co. KG, Germany) and then dried for 12 h at a thermostat setting of 37°C. The samples were then deparaffinized with xylene and dehydrated with a series of ethanol reductions. After dehydration, the samples were placed in a 65°C buffer solution at pH 9.0 (Target retrieval solution, pH 9, Dako, Agilent Technologies, USA) and treated twice with a break of 70°C in a microwave at 450 W for 5 min to release epitopes to bind the antigen to the antibody. The samples were then cooled at room temperature (~22°C) and amplified for 5 min with endogenous peroxidase blocking reagent (Dako Endogenous Enzyme Block, Agilent Technologies, USA) to block endogenous peroxidase. Rat and mouse polyclonal antibodies (H-031-31, Phoenix Pharma Inc., USA) at a dilution of 1:500 was used as primary antibodies. For IR cell and primary antibody responses, the antigen-antibody complex was labeled by application of the secondary antibody for 30 min and then stained by application of the DAB+ complex for 5 min (Dako REAL™ EnVision™ Detection System, Agilent Technologies, USA). The tissues were stained with hematoxylin to provide contrast to the sample and to avoid artifacts. The stained samples were incubated in a humid chamber at ~22°C and the samples were rinsed with phosphate buffer (Wash Buffer, pH 7.4, Dako, Agilent Technologies, USA).

Histological samples of the pylorus of the dog’s stomach were used as a positive control for ghrelin antibodies. The same samples were used as negative controls, except that they were treated with an antibody diluent instead of the primary antibody (no immunoreaction was observed).

The quantitative composition of IR cells was evaluated in each sample in 10 fields of vision by determining the mucosal area and the number of positive cells in it by determining the number of IR cells in 1 mm^2^. The samples were examined at 400× with an EVOS M5000 microscope (Invitrogen™, USA).

### Statistical analysis

The assumption of normal data distribution was assessed using the Shapiro-Wilk test and visual inspection of their histograms and normal Q-Q plots. The assumption of homogeneity of variances was tested by Levene’s test. To determine whether there were any statistically significant differences between the three independent groups, we used the Kruskal-Wallis H test with pairwise comparisons using Dunn’s procedure [33] with a Bonferroni adjustment. To determine whether there were any statistically significant differences between the two groups, we used the Mann-Whitney U-test or the independent samples t-test. Measurement of the strength and direction of the association between two continuous or ordinal variables was evaluated by Spearman rank order correlation. These tests were conducted using Statistical Package for the Social Sciences Statistics version 22 (IBM Corporation, Chicago, Illinois). All statistical analyses were performed at p=0.05 and data are presented as mean ± standard deviation unless otherwise stated.

## Results

**Figure-1:**
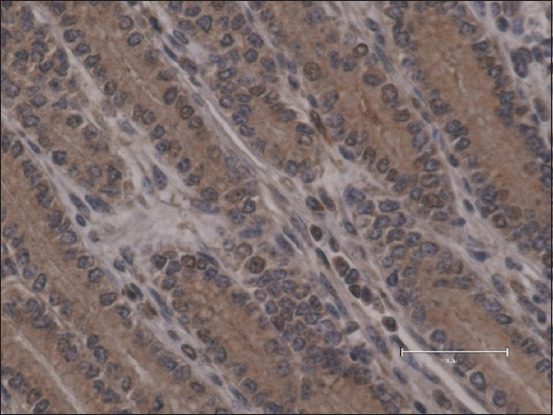
Abomasum *pars pylorica* in a 13-week-old calf from the control group – no ghrelin immunoreactive + cells observed (400×, DAB + hematoxylin, scale bar 50 μm).

Examination of samples from different parts of the gastrointestinal tract showed that ghrelin-IR cells were more abundantly localized in the cytoplasm of the abomasum muscle gland cells in the *pars fundalis* and *pars pylorica*, and to a lesser extent in the duodenum and jejunum. In the area of the fundic glands, as well as in the duodenum and jejunum, ghrelin-IR cells were found mainly at the apical ends of the glandular cells, but in the area of the pyloric glands, these cells were mainly found in paranuclei and also in the cell nuclei. The ghrelin-IR cells in the samples stained brown when stained with DAB+, and the cells themselves were round, oblong, and square in shape. No positive staining results were observed in the negative control samples (Figure-1).

**Figure-2:**
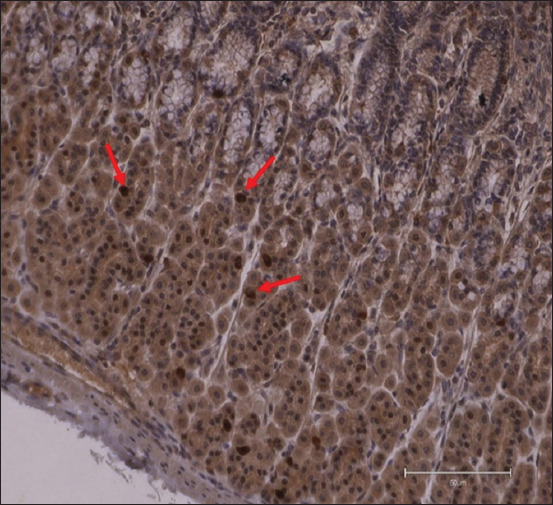
Ghrelin immunoreactive + cells (arrows) in a 13-week-old calf from the control group in the abomasum *pars fundalis* (200×, DAB + hematoxylin, scale bar 50 μm).

**Figure-3:**
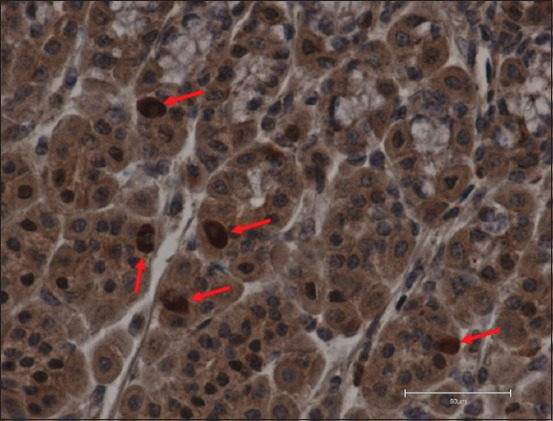
Ghrelin immunoreactive + cells (arrows) in a 13-week-old calf from the control group in the abomasum *pars fundalis* (400×, DAB + hematoxylin, scale bar 50 μm).

**Figure-4:**
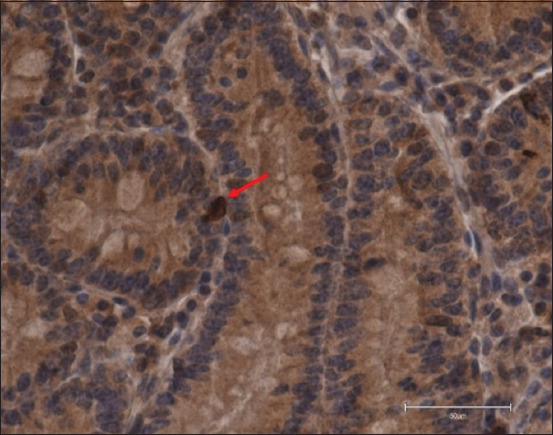
Ghrelin immunoreactive+cell (arrow) in a 13-week-old calf from the middle of the duodenum of a control group (400×, DAB + hematoxylin, scale bar 50 μm).

**Figure-5:**
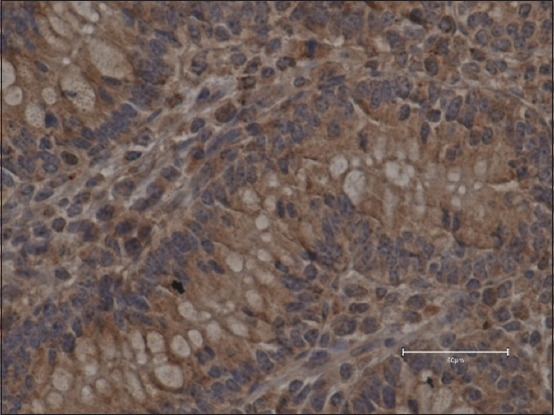
Middle part of the jejunum in a 13-week-old calf from the prebiotic group – no ghrelin immunoreactive + cells observed (400×, DAB + hematoxylin, scale bar 50 μm).

The examination of histological samples showed that the number of ghrelin-IR cells in the abomasal fundic gland area was significantly higher in the CoG (Figures-2 and 3), than in the prebiotic group (PreG) and SynG (p=0.0001), while the difference between the PreG and SynG was not significant (p=0.700). No other significant differences were found for the number of ghrelin-IR cells in the abomasum *pars pylorica*, the duodenum (Figure-4), and the middle of the jejunum (Figure-5 and Table-2).

**Table 2:** Number of ghrelin-IR-positive cells in the abomasum and intestine of 1 mm^2^ in calves after 56 days of feeding Jerusalem artichoke flour concentrate and its synbiotic with yeast *Saccharomyces cerevisiae*.

Localization of ghrelin IR+cells	The number of ghrelin IR+cells in the group (n=9)						p-value, Kruskal–Wallis test

	CoG		PreG		SynG		
		
	Me	Q1-Q3	Me	Q1-Q3	Me	Q1-Q3	
Abomasum *pars fundalis*	12^a^	10-14	6^b^	5-9	5^b^	4-8	0.0001
Abomasum *pars pylorica*	2	0.75-3	1.5	1-2	1	0-2	0.289
Middle part of the duodenum	1	0-2	0.5	0-1	1	0-2	0.098
Middle part of the jejunum	1	0-1	0	0-1	0	0-1	0.222

^a,b^ Values on the same line without any common letter are significantly different. IR=Immunoreactive, CoG=Control group, PreG=Prebiotic group, SynG=Synbiotic group

The live weight of the slaughtered calves on day 56 of the study was 115.3±21.73 kg in CoG, 130.0±17.32 kg in PreG, and 119.0±7.94 kg in SynG. There was no correlation between the number of ghrelin-IR cells found in the digestive tract of calves and the live weight of calves on day 56 of the study (r=0.025, n=9, and p=0.948).

## Discussion

Ghrelin is a hormone that plays an important role in the regulation of various physiological processes. It affects the growth and development of the body by promoting the release of growth hormone from the pituitary gland [19,21] and, at the same time, regulates hunger and satiety [24,25].

In ruminants, ghrelin is mainly produced in endocrine cells of the abomasal mucosa [28]. In the stomach, ghrelin-containing cells are found in greater amounts in the fundic gland area and to a lesser extent in the pyloric gland area. Ghrelin-producing cells are also found in the intestines but are less abundant than in the abomasum and gradually decrease from the duodenum to the colon [21]. In this study, it was also found that the number of ghrelin-IR cells in the fundic gland area of the abomasum was higher in both control and feed additive supplemented calves than in the pyloric gland area, and the number of these cells was significantly lower in the middle part of the duodenum and jejunum.

In a study of 2-month-old sheep (n=16) with the same diet but different feeding regime (Group 1 – *ad libitum*, Group 2 – *ad libitum* and intravenous ghrelin injection (1 g/kg), Group 3 – fed once per day, and Group 4 – fed twice a day), which lasted for 1 month, it was observed that the number of ghrelin-IR cells in the areas of the cardiac, fundic, and pyloric glands of the abomasum did not differ significantly among groups of sheep with different feeding regimens [34]. In our study, all groups of calves had the same standard diet, only the added feed additives differed. Thus, it can be assumed that it was the feed additives that affected the number of ghrelin-IR cells. In the abomasum, duodenum, and jejunum, the ghrelin-IR cells were higher in the controls compared to calves fed with Jerusalem artichoke flour concentrate or this flour combined with yeast *S. cerevisiae* strain 1026. For example, in the calves of the CoG, the number of ghrelin-IR in the fundic gland zone of the abomasum was Me=12, IQR 10-14, in PreG Me=6, IQR 4-9, but in SynG Me=5, IQR 5-8. A higher number of ghrelin-IR cells in the gastrointestinal tract could indicate a higher amount of ghrelin secretion. Considering that ghrelin is produced in response to hunger and decreases after a meal when the animal experiences satiety [26,27], it can be assumed that the calves of the CoG experienced greater hunger than the calves received feed additives. Many studies have shown that different feed additives improve the digestibility of nutrients in animals so that the same dose of feed keeps the animals feeling full and improves the usability of the food they have ingested. For example, Sullivan and Martin [35] reported that the addition of yeast *S. cerevisiae* to cow diets improved cellulose digestibility, while Samanta *et al*. [36] found that the addition of prebiotic fructooligosaccharide to the diet significantly improved the digestibility of organic matter and dry matter in the rumen.

Very limited information is available on the modification of the number of ghrelin-IR cells due to feed intake. However, in a study in rats, the number of ghrelin-IR cells in the stomach increased significantly after 7 days of fasting, but the number of active cells decreased to normal levels after feed intake [30].

Lower live weight could also indicate greater hunger and poorer digestibility of consumed feed or lower feed intake over time. Although the live weight of calves in the CoG s (115.3±21.73 kg) was lower than that of calves which additionally were supplemented with feed additives (PreG 130.0±17.32 kg and SynG 119.0±7.93 kg), no correlation was found between the number of ghrelin-IR cells in different parts of the digestive tract and the live weight of calves on day 56 of the study (r=0.025, p=0.948).

However, to better assess the changes in the amount of ghrelin-IR cells depending on the feed additives, studies with larger groups of calves are required. The amount of ghrelin in the blood plasma should also be determined, which could provide more accurate information on the hormonal regulation of hunger and satiety, as well as information on the effects of ghrelin on animal growth.

## Conclusion

The addition of Jerusalem artichoke flour (12 g) containing 5 g of prebiotic inulin and its combination with the yeast *S. cerevisiae* strain 1026 (5 g) in calves resulted in a lower number of ghrelin-IR cells in the abomasum and intestines and, although insignificantly, increased live weight (p=0.491), suggesting that calves in these groups with the same feed intake as the CoG had a better breakdown of nutrients, thus having a longer feeling of satiety. The limitation of this study was the small number of calves. We followed the 3Rs principle – reduce the number of animals used per experiment or study consistent with the scientific aims. This was discussed with the Council for Ethical Treatment of Animals, Latvia University of Life Sciences and Technologies, and it was decided to use only five animals per group and slaughter only three animals per group, as this was estimated to be a sufficient number of animals to meet our research goals. Statistical analysis of the data was performed using methods suitable for a small amount of data.

## Authors’ Contributions

SJ: Collected the samples, performed the clinical examination of calves, evaluated immunohistochemical staining of histological specimens, and drafted and revised the manuscript. AI: Designed the concept for this research and scientific paper. AI*: Collected the samples and performed the clinical examination of calves. MZ: Performed the statistical analysis of all data. LG: Collected the samples and performed the clinical examination of calves. All authors read and approved the final manuscript.
